# Lichen Biomonitoring of Airborne Microplastics in Milan (N Italy)

**DOI:** 10.3390/biology11121815

**Published:** 2022-12-14

**Authors:** Mehriban Jafarova, Tania Contardo, Julian Aherne, Stefano Loppi

**Affiliations:** 1 Department of Life Sciences, University of Siena, 53100 Siena, Italy; 2 School of Environment, Trent University, Peterborough, ON K9L 0G2, Canada

**Keywords:** atmospheric deposition, biomonitoring, lichen, microplastics, microfibres, Milan

## Abstract

**Simple Summary:**

The atmospheric deposition of microplastics (MPs) is a growing concern for human health. In this research, we studied the deposition of airborne MPs in urban areas of Milan and a remote area in North Italy using lichen transplants. The results demonstrate that lichens are suitable biomonitors for studying airborne MPs. The findings of airborne MPs, even in remote areas, indicate the inevitable exposure of humans and, therefore, potential health risks.

**Abstract:**

This study investigated the deposition of airborne microplastics (MPs) in the urban area of Milan across 12 sites and at a background control site (northern Italy) using 3-month transplants of the fruticose lichen species *Evernia prunastri* (exposed in triplicate). The primary objective was to evaluate the use of lichen transplants for the assessment of MP deposition; as such, the study sites spanned a gradient in vehicular traffic and population density across four concentric land-use zones (i.e., urban parks, centre, semi-periphery, and periphery). A total of 149 MP particles were detected in the exposed lichen samples; 94.6% were classified as fibres and 5.4% as fragments. The control site and urban parks experienced a similar number of MPs per gram of dry lichen (20–26 MP/g), while a higher number of MPs were detected in central and peripheral areas (44–56 MP/g), with a clear increasing gradient from the city centre towards the periphery. We estimated the MP deposition in Milan to be in the range of 43–119 MPs m^2^/d, indicating that people living in Milan are exposed to airborne MPs, with potential health effects. This study suggests that lichens are suitable biomonitors of airborne MPs under a relatively short exposure of three months in urban environments.

## 1. Introduction

Environmental contamination by microplastics (MPs), i.e., plastic particles ≤ 5 mm in length, is a well-known worldwide phenomenon, which has been reported across every environment, including remote areas [1,2]. MPs are both manufactured directly in micro-forms (e.g., as microbeads in personal care products), called ‘primary’ microplastics, or are the product of the degradation of larger plastic objects, called ‘secondary’ microplastics [3]. MPs enter the environment in different ways (e.g., through the release of wastewater, wind dispersal of degraded fragments, mismanagement of plastic wastes, etc.) and their slow degradation makes them very persistent [4,5]. The toxicity of MPs is far from fully understood due to their heterogeneous polymer composition; nonetheless, several authors have shown that MPs can adsorb organic and inorganic pollutants [6,7], and pathogens [8], possibly contributing to their dispersion in the environment, their accumulation in the trophic chain, and their enhanced toxicity [9,10].

To date, the majority of studies have focused on aquatic (primarily marine) environments, while compartments such as the atmosphere are understudied [11]. However, evidence is quickly growing about the presence of MPs (in particular, microfibres (mf) from textiles) in the atmospheric fallout of urban and remote areas [2,12,13]. The persistence and fate of atmospheric MPs are strictly related to polymer density, shape, and size, as well as to climatic factors, population density, and air turbulence in urban topographies [14]. Suspended MPs can be easily inhaled and ingested, and even though studies on human health outcomes are few, the first findings show that MPs can cause abrasion and inflammation, as well as alteration of the gut microbiome, among others [15,16]; MPs have also been observed in human blood [17]. To better understand the health risks, it is of paramount importance to measure the exposure to MPs by quantifying their occurrence, especially in the urban atmosphere, as urban environments host the vast majority of the human population [18,19]. Furthermore, urban environments are dominant sources of microplastics in remote environments.

The use of living organisms, especially lichens, has proven to be widely effective in air pollution monitoring of potentially toxic elements (PTEs), providing an assessment of their deposition and biological effects [20,21]. The use of lichen transplants is a well-established and cost-effective technique to monitor air pollution since these organisms can be easily collected in an unpolluted area and transplanted to a large number of sites across a chosen study area, thus providing a dense network of monitoring stations, that cannot be realized with traditional monitoring devices that are expensive and sparsely distributed [22].

Only one study has been carried out using lichens as bioaccumulators of airborne MPs [23], it showed that the lichen *Flavoparmelia caperata* naturally growing in the surroundings of a landfill dumping site in central Italy was an effective biomonitor of the deposition of MPs released from the landfill. The aim of the current study was to evaluate the use of lichen transplants to measure the deposition of airborne MPs in urban areas, using Milan (northern Italy) as the pilot study area. To the best of our knowledge, this is the first study to use the lichen transplant technique for the assessment of atmospheric MP deposition, and the first MP biomonitoring study in an urban environment.

## 2. Materials and Methods

### 2.1. Study Area

**Figure 1:**
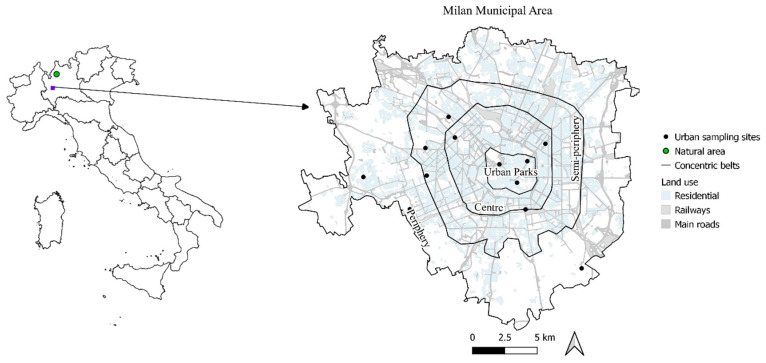
Map showing the location of the study area in northern Italy (**left**), the location of sampling sites (n = 12) in Milan (**right**), and the background control site (**left**).

This study was carried out in the urban area of Milan, northern Italy (Figure 1). Milan is among the most culturally important Italian cities, and it plays a key role in the national economy. The city is home to 1.4 million inhabitants, which doubles during the daytime hours for work reasons, and covers a surface area of 181 km^2^. Milan is embedded in the broader region of the Po Plain, which is characterized by intense industrial activity, high vehicular traffic, and acute population density. The area is also characterized by air stagnation and frequent thermal inversions that entrap air pollutants making the Po Plain one of the most polluted areas in Europe, especially for particulate matter [24].

### 2.2. Experimental

The epiphytic (tree-inhabiting) lichen *Evernia prunastri* was selected as the test species in this pilot study due to its well-known capacity for bioaccumulating trace elements [22], its high surface–volume ratio, and its shrubby shape that potentially improves the entrapping of MPs. The lichen material was collected from tree branches in a remote area of Tuscany, central Italy (see [25]). The material was then transplanted to the urban area of Milan following a stratified random sampling design based on land use and distance from the city centre (Figure 1). The main core of the city is mostly pedestrian, with urban parks and traffic restrictions, while in the periphery land use shifts from residential to industrial/agricultural with no traffic restrictions. According to these differences, four concentric zones were established (Urban Parks, Centre, Semi-periphery, and Periphery), and three sampling sites were randomly selected in each zone. Further, triplicate lichen samples were transplanted to each sampling site and deployed at a height of 1.5–2 m tied to the branches of trees. A control site was selected about 50 km north of Milan, in a mountainous region far from any local sources of air pollution, where again three lichen samples were transplanted. The exposure lasted three months (December 2018 to February 2019), which is regarded as optimal for this species [22]. For further details on the study design, see [25].

### 2.3. Microplastic Analysis

In the laboratory, air-dried (residual water < 10%) samples were individually digested using a wet peroxide oxidation method [23,26]. Samples were then vacuum filtered onto cellulose filter papers (Watman Grade 1, 1001-090, 11 µm), dyed with 2 mL of Rose Bengal (4,5,6,7-tetrachloro-2′,4′,5′,7′-tetraiodofluorescein, 200 mg/L) to help visually differentiate synthetic material from organic matter, and the filters placed into glass Petri dishes for storage. The filter papers were examined for MPs under a stereomicroscope (Eurotek OXTL101TUSB equipped with an MDCE-5C digital camera) following a five-criteria method [23,26]. Microfibres and fragments that met at least two of the criteria, and were not stained by Rose Bengal, were considered anthropogenic and photographed [27], and further verified using a hot needle test [28,29]. All plastic microfibres and fragments were measured using the open-source image processing software ImageJ. To control for any laboratory contamination, air exposure blanks and analytical process blanks were routinely run during the analysis. Overall, 39 lichen samples (13 sites with triplicate samples) were analysed individually.

### 2.4. Estimation of MP Deposition Rates

The number of MPs accumulated by lichens can be converted into MP deposition rates. To do this, a mass/area ratio for *E. prunastri* of ~160 g/m^2^ was calculated by cutting several thallus pieces and measuring their surface area and dry weight [22]. Based on the exposure period of three months, MP accumulation in lichens was converted into estimates of average daily MP deposition, according to the formula:

MP deposition (MPs/m^2^/d) = MP accumulation (MPs/g) × 160 (g/m^2^)/90 (days).

### 2.5. Statistical Analysis

Data normality was verified with the Shapiro–Wilk test. Significant differences between sampling zones were evaluated by one-way ANOVA followed by the LSD test for post hoc comparisons. A probability of 0.05 was chosen as the level of significance for all statistical tests, and all statistical analyses were carried out using the open-source software R [30].

## 3. Results

A total of 149 MP particles were detected across the thirteen study sites; 94.6% were classified as fibres, while just 5.4% as fragments. Fragments were detected at all sites except for the background control site, and their size was in the range of 127–615 µm, with a mean length of 354 µm.

At the control site, an average of 20 MPs per gram of dry lichen were found, which was significantly (*p* < 0.05) different (lower) compared with all urban sites except for parks (Table 1). A gradient was evident from the city centre towards the periphery, with increasing bioaccumulation of MPs, from 44 to 56 MPs per gram of dry lichen (Table 1). The conversion of MP accumulation in lichens into estimates of average daily MP deposition rates, showed a range for Milan of 43–119 MPs/m^2^/d (Table 1).

**Figure 2:**
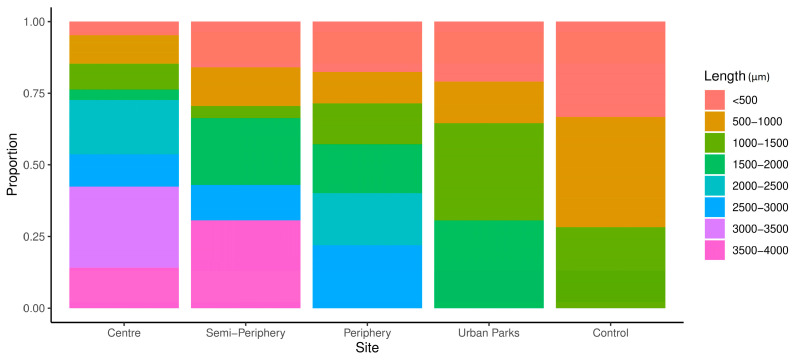
Fractional distribution of the length (µm) of microfibres (n = 141) across the four urban sampling zones in Milan and the background control site in northern Italy.

The length of fibres did not show any trend nor statistically significant differences between sites, but the shortest fibres were found at the control site (Table 1). The distribution of fibre length ranged from 83 µm to 3980 µm, with a range of 181–1397 µm at the control site, 128–1839 µm in urban parks, 111–3980 µm in the city centre, 109–3624 µm in the semi-periphery, and 83–2997 µm in the periphery; the longest fibres (3500–4000 µm) were observed in the city centre and in the semi-periphery (Figure 2).

## 4. Discussion

Our results show that, after three months of exposure, samples of *E. prunastri* efficiently allow the detection of spatial trends in the accumulation of airborne MPs in an urban environment. Although shorter and longer exposure periods may be similarly suitable, three months are known to be optimal for the bioaccumulation of PTEs for this lichen species [22], as well as for most lichens used in similar transplant studies [31,32,33,34].

At the control site, located in a mountainous region about 50 km north of Milan and far from sources of MP pollution, we found an accumulation of airborne plastic microfibres (12–24 mf/g), similar to urban parks (20–24 mf/g) in Milan, a large city with 1.4 million inhabitants. However, these results are consistent with the 18–23 mf/g found in lichens from a control site in central Italy [23], and with the 15–30 mf/g found in moss samples collected at remote sites in Ireland [26].

Based on these results, we could speculate that airborne microfibres have reached a constant baseline concentration and deposition rate, at least in Europe; however, it should be considered that in the present study we refer to plastic microfibres, while the proportion of anthropogenic fibres that were plastic in the studies of [23,26] was about 25%, leading to estimates of ca. 4–8 plastic mf/g, i.e., three times less than in the present study. Hence, we suggest that remote areas of northern Italy are more polluted than remote areas of central Italy, as well as remote areas of Ireland, perhaps because the former is downwind of large urban and industrialized areas in the Po Plain.

The trend of increasing concentration of MPs from the city centre towards the periphery sharply contrasts with the decreasing bioaccumulation of PTEs found in the same area by [25]. These results suggest that sources and deposition of airborne PTEs and MPs are different or driven by different factors and that lichens can effectively be used to detect both types of contamination. Nonetheless, the fact that the number of MPs per gram of dry lichen increased from the urban centre to the peripheral zones, compared with the control site, which was much lower, is strong support of a local origin of MPs, at least most (55–65%) of them.

Although the dynamics of MP accumulation and release from lichen samples, as well as the role of thallus morphology and growth during the exposure period, have not yet been investigated, assuming no release and a constant accumulation throughout the three months of exposure (and using a mass/area ratio of 160 g/m^2^ for *E. prunastri*), we estimated that MP deposition in Milan was in the range of 43–119 MP/m^2^/d. This value is probably an underestimate given potential release or inefficient accumulation, nonetheless, it is in the range of reported values for several urban areas [35]. The estimated average daily atmospheric deposition of plastic microfibres in Milan excluding urban parks (76–93 mf/m^2^/d) is, however, higher than the 1–44 mf/m^2^/d reported for several areas worldwide (see Table 3 in [13]). Nevertheless, at the control site, we estimated a deposition of 21–43 mf/m^2^/d, which is similar to the 15–32 mf/m^2^/d reported for Paris [36]. It is noteworthy that our lichen samples were exposed at 1.5–2 m from the ground, which is the height of human respiration [37]; thus, our results provide an estimate of daily MP exposure for humans. The presence of MPs in the air is a health concern as MPs can reach deep into the lungs [16] and cause inflammation [38]. However, the MPs observed in this study are coarse (minimum length of about 80 µm) compared to the particles that are likely to penetrate deeply into the respiratory system (MPs < 2.5 µm in diameter). It remains to be investigated if and how lichens can accumulate plastic of small diameter, e.g., nanoparticles, although there is no reason to assume the contrary.

In general, fibres are more prone to long-range transport in comparison with other types of MPs, due to differences in weight and density, since heavier fragments tend to settle more easily [39]. Moreover, fibres have a greater surface area-to-volume ratio, which increases drag force and reduces settling velocity [40]. In the present study, the number of plastic fibres was much higher than fragments at all sites, and no plastic fragments were observed at the background control site. The presence of MPs in remote areas is mainly attributable to the long-range circulation of air masses [2]. Further, our results are consistent with the finding that the majority of MPs were fibres in the atmospheric fallout of Paris, France [12,36]; London, England [39]; Dongguan, China [41]; and Gdansk, Poland [42].

On average, the control site experienced the shortest fibre length and the centre of Milan the longest. A similar higher proportion of shorter microfibres at sites more remote from urban centres has been reported elsewhere [29]. These observations are consistent with the fact that smaller MPs are dispersed over longer distances.

## 5. Conclusions

This is the first study to use the lichen transplant technique to assess the atmospheric deposition of microplastics in an urban environment. Our results suggest that lichens can help to detect the deposition of airborne MPs under a relatively short exposure of three months. Further, our findings indicate that the inhabitants of Milan are exposed to airborne plastic microfibres, and the potential health effects should be investigated. Lastly, the abundance of plastic microfibres in a remote mountainous area far from Milan, indicated that MPs are subjected to long-range transport. Future studies should assess the polymer type and age of MPs, especially with respect to potential impacts on human health.

Although the present findings are based on one lichen species and a single exposure period, they are nonetheless important for environmental decision- and policymakers who require effective research and monitoring strategies to support meaningful actions on microplastic pollution.

## Figures and Tables

**Table 1:** Mean (±standard error) number of MPs accumulated by the fruticose lichen *Evernia prunastri* in each concentric zone after three months of exposure (number per gram of dry weight), along with the proportion of fibres and fibre length in each zone. Different letters indicate statistically significant differences (*p* < 0.05) in MP accumulation between zones. The range of estimated deposition of MPs in each zone is also reported.

Summary Variable (Unit)	Control	Urban Parks	Centre	Semi-Periphery	Periphery
Lichen samples (n)	3	9	9	9	9
MPs (nr/gr dw)	20 ± 4 c	26 ± 1 c	44 ± 1 b	48 ± 3 ab	56 ± 5 a
Fibres (%)	100 ± 0	91 ± 5	97 ± 3	95 ± 2	93 ± 5
Fibre length (µm)	616 ± 92	867 ± 146	1076 ± 156	951 ± 143	885 ± 117
Deposition (MPs/m^2^/d)	21–43	43–50	75–82	76–91	91–119

## Data Availability

Not applicable.
